# The Impact of the COVID-19 Pandemic on the Mental Health of Older Primary Care Patients and Their Family Members

**DOI:** 10.1155/2022/6909413

**Published:** 2022-10-15

**Authors:** Tara Seibert, Matthew W. Schroeder, Anthony J. Perkins, Seho Park, Eleanor Batista-Malat, Katharine J. Head, Tamilyn Bakas, Malaz Boustani, Nicole R. Fowler

**Affiliations:** ^1^Indiana University School of Medicine, Indianapolis, IN 46202, USA; ^2^Indiana University Center for Aging Research, Indianapolis, IN 46202, USA; ^3^Regenstrief Institute Inc., Indianapolis, IN 46202, USA; ^4^Department of Biostatistics and Health Data Science, Indianapolis, IN 46202, USA; ^5^Indiana University-Purdue University Indianapolis, Department of Communication Studies, Indianapolis, IN 46202, USA; ^6^University of Cincinnati College of Nursing, Cincinnati, OH 45219, USA; ^7^Center for Health Innovation and Implementation Science, Indiana Clinical and Translational Science Institute, Indianapolis 46202, USA

## Abstract

The COVID-19 pandemic introduced mandatory stay-at-home orders and concerns about contracting a virus that impacted the physical and mental health of much of the world's population. This study compared the rates of depression and anxiety in a sample of older primary care patients (aged ≥65 years old) and their family members recruited for a clinical trial before and during the COVID-19 pandemic. Participants were dyads enrolled in the Caregiver Outcomes of Alzheimer's Disease Screening (COADS) trial, which included 1,809 dyads of older primary care patients and one of their family members. Mean scores on the Patient Health Questionnaire-9 (PHQ-9) and the Generalized Anxiety Disorder Scale-7 (GAD-7) were measured and compared before and during the pandemic. We found no difference in depression and anxiety among dyads of older primary care patients and their family members recruited before and during COVID-19. Additionally, we found that older primary care patients and family members who reported their income as comfortable had significantly lower depression and anxiety compared to those who reported having not enough to make ends meet. Along with this, older primary care patients with a high school education or less were more likely to have anxiety compared to those with a postgraduate degree. Moreover, our findings support the notion that certain demographics of older primary care patients and family members are at a higher risk for depression and anxiety, indicating who should be targeted for psychological health interventions that can be adapted during COVID-19. Future research should continue monitoring older primary care patients and their family members through the remainder of the COVID-19 pandemic.

## 1. Introduction

In March 2020, the World Health Organization (WHO) officially classified COVID-19 as a global pandemic [1]. Countries and territories across the world began to implement quarantine and shelter-in-place orders in an attempt to limit social contact and slow the spread of COVID-19. By April 2020, more than 90% of United States residents were under a stay-at-home order [2]. Despite these measures, COVID-19 cases in the US continued to rise, totaling more than 1.7 million by the end of May 2020 [3]. Adults aged ≥65 years old were identified as especially high-risk, with the highest rate of COVID-19-associated hospitalizations, the most severe complications, and the highest mortality rate [4]. In March and April 2020, nearly 80% of US deaths attributed to COVID-19 occurred in adults aged ≥65 years old [5]. One study of older adults (aged ≥60 years old) reported that 58.1% of them were afraid of contracting COVID-19 and 43.5% feared losing their life due to COVID-19 [6]. Considering the increased morbidity and mortality from COVID-19 in older adults, in combination with mandatory social isolation secondary to stay-at-home orders, it is necessary to explore the potential negative impact of COVID-19 on the psychological health of this population.

Before COVID-19, national representative samples of older adults aged ≥65 years old experienced symptoms of depression and anxiety at around 18.4% and 11.2%, respectively [7, 8]. Research conducted by the World Health Organization (WHO) found increased incidences of mental health struggles in the global population during COVID-19, with rates of depression at around 28.0% and anxiety at around 26.9% among all age groups [9]. Additionally, recent studies have delineated that there are certain demographics whose mental and psychological health has been disproportionately impacted by COVID-19. These studies found that the highest rates of psychological distress were among individuals aged 18–30 and ≥60 years old, females, those with higher levels of education, and migrant workers [10]. By November 2020, rates of anxiety and depression were four times higher than national rates from early 2019, showing that the prevalence of mental health disorders is growing despite the pandemic. The results from these studies demonstrate increasing rates of anxiety and depression, secondary to COVID-19, and support the theory that certain subgroups may be disproportionately impacted by the changes brought on by COVID-19.

The combination of COVID-19, depression, and anxiety not only affects older adults but also potentially their family members, given the interdependence of emotional and psychological well-being within families. With stay-at-home orders in place, the burden and stress levels of family members of older adults have likely increased [11]. A recent study conducted during the COVID-19 pandemic reported significantly higher rates of adverse mental and psychological health conditions among family caregivers, including increased fear, anxiety, frustration, and depression due to COVID-19 [12]. Family caregivers who reported delaying medical care for their relative or taking efforts to reduce contact with other people due to COVID-19 were more likely to report experiencing psychological health symptoms and decreased well-being [11]. While these studies provided insight into how COVID-19 has affected family caregivers of older adults, no research has explored its effect on family members of older primary care patients who would potentially provide care if the patient became ill and needed it.

The objective of this study was to measure and compare the rates of depression and anxiety in two cohorts of dyads of older primary care patients and their family members who were recruited before and during the COVID-19 pandemic.

## 2. Methods

### 2.1. Setting, Participants, and Study Design

Secondary data analysis was conducted using baseline data from the ongoing Caregiver Outcomes of Alzheimer's Disease Screening (COADS) study, a randomized controlled trial measuring the benefits, risks, and harms of Alzheimer's disease and related dementias (ADRD) screening among older adults in primary care and their family members [13]. COADS recruitment occurred from October 2018 to September 2021. Dyads of older primary care patients and one of their family members were enrolled in person in their primary care office or via telephone and provided informed consent to participate in the study. Study approval was granted by the Institutional Review Board at Indiana University.

Patients were eligible if they were aged ≥65 years old, had at least one visit to their primary care physician in the past 12 months, could provide informed consent, were able to communicate in English, and had no previous or existing diagnosis of Alzheimer's disease, related dementia, or mild cognitive impairment [13]. Patients were excluded from the study if they were permanent residents of a nursing facility, had a serious mental illness (such as bipolar disorder or schizophrenia), or had been given a prescription for a cholinesterase inhibitor or memantine. Family member participants were eligible if they were aged ≥21 years old, identified by the patient as the person most likely to provide care for the patient if the patient needed care, could provide informed consent, could communicate in English, and lived within a 50-mile radius of the patient. Nonfamily members, such as close friends, were eligible for inclusion in the study as long as they met all family member inclusion criteria and were designated as the healthcare power of attorney.

### 2.2. Measures

The study used the Patient Health Questionnaire (PHQ-9) to assess symptoms of depression for the entire sample. The PHQ-9 is a valid and reliable measure of symptoms of depression in older adults and informal caregivers [14, 15]. It is a 9-item questionnaire developed from the Primary Care Evaluation of Mental Disorders Patient Health Questionnaire (PRIME-MD PHQ) based on the DSM-IV criteria. The sensitivity and specificity are 88% and 88%, respectively [14]. The questionnaire asked respondents to consider how often they have been bothered by various symptoms of depression in the past two weeks, with scores ranging from 0 “not at all” to 3 “nearly every day.” The total score is representative of the respondent's severity of depression. The Generalized Anxiety Disorder Scale (GAD-7) was used to measure anxiety and it is a valid and reliable screening tool for recognizing generalized anxiety disorders in an adult primary care population [16]. The sensitivity and specificity are 89% and 82%, respectively. The questionnaire asks respondents to report how often they have been bothered by various symptoms of anxiety in the past two weeks, with scores ranging from 0 “not at all” to 3 “nearly every day.” The total score is used to screen for the presence and severity of a generalized anxiety disorder.

### 2.3. Independent Variables and Covariates

The primary independent variables were depression and anxiety, measured in the two cohorts. Patients and family members recruited “before COVID-19” included baseline PHQ-9 and GAD-7 scores from the dyads enrolled from October 2018 to March 10, 2020. Patients and family members recruited “during COVID-19” included baseline PHQ-9 and GAD-7 scores from dyads recruited from March 11, 2020, to September 30, 2021 [17], which aligns with the stay-at-home orders for the state of Indiana. COVID-19 data collection continued until September 30, 2021, which was the date our final participant completed their baseline assessment. Covariates for each member of the dyad included age, self-identified sex and race, education level, the perceived comfort of their income to meet their needs, and primary care clinic location.

### 2.4. Statistical Analysis

We used two-sample *T*-tests and Chi-square tests to compare the demographic variables between patients and their family members recruited before COVID-19 versus during COVID-19 cohorts. Differences between those recruited before COVID-19 and during COVID-19 cohorts in rates of depression and anxiety were analyzed as well. We conducted a logistic regression to assess the relationship between the two cohorts with depression and anxiety after adjusting for demographic characteristics. All analyses were conducted using SAS v9.4 (Cary, NC).

## 3. Results

Characteristics of patients and family members are shown in Table 1. Our total study sample consisted of 1,809 dyads of older adults and family members. Of these, 1,118 dyads were evaluated before COVID-19, and 691 dyads were evaluated during COVID-19. Characteristics were similar between participants recruited before COVID-19 and during COVID-19 groups apart from age, race, perceived income, educational level, relationship to patient, primary care clinic location, and patient depression. The mean age of patients recruited before COVID-19 and during the COVID-19 groups was 73.8 and 73.6 years old, respectively. The mean age of the family members recruited before COVID-19 and during the COVID-19 groups was 63.6 and 65.1 years old, respectively. Compared with those recruited before COVID-19, a smaller proportion of participants recruited during COVID-19 was African American.

We examined rates of depression and anxiety for patients while adjusting for their characteristics at a significance level of 5% (Table 2). We found no significant difference in depression (AOR = 0.84, 95% CI [0.67–1.06]) or anxiety (AOR = 0.85, 95% CI [0.62–1.17]) between patients recruited before and during COVID-19. Male patients were less likely to have depression than female patients (AOR = 0.78, 95% CI [0.62–0.97]). Patients with a high school degree or less (AOR = 2.13, 95% CI [1.35–3.35]) and some college or college degree (AOR = 1.48, 95% CI [1.01–2.17]) were more likely to have anxiety than patients with a postgraduate degree. Patients with a comfortable income (AOR = 0.21, 95% CI [0.11–0.37]) or just enough to make ends meet (AOR = 0.43, 95% CI [0.23–0.80]) were less likely to have depression than patients who reported having not enough to make ends meet. Similarly, both patients with a comfortable income (AOR = 0.23, 95% CI [0.13–0.44]) and just enough to make ends meet (AOR = 0.44, 95% CI [0.23–0.85]) were less likely to have anxiety than those with not enough to make ends meet. Interestingly, patients recruited from rural primary care clinical locations were less likely to have depression than those recruited from urban primary care clinic locations (AOR = 0.72, 95% CI [0.52–1.00]). After conducting a sensitivity analysis to include if a patient ever had COVID-19 or lost a family member, friend, or neighbor to COVID-19, we found no difference in depression and anxiety between patients recruited before and during COVID-19 (not shown in tables).

We examined rates of deperession and anxiety for family members while adjusting for their characteristics at the significance level of 5% (Table 3). There was no significant difference in depression (AOR = 1.18, 95% CI [0.92–1.52]) and anxiety (AOR = 1.04, 95% CI [0.78–1.40]) between family members recruited before or during COVID-19. Increased family member age was associated with decreased odds of depression (AOR = 0.99, 95% CI [0.98–1.00]) and anxiety (AOR = 0.97, 95% CI [0.96–0.98]). African American family members were less likely to have depression (AOR = 0.52, 95% CI [0.35–0.78]) and anxiety (AOR = 0.57, 95% CI [0.37, 0.89]) compared with white family members. Family members with a high school degree or less were more likely to have depression than those with a postgraduate degree (AOR = 1.47, 95% CI [1.01–2.15]). Family members who reported having a comfortable income had significantly lower rates of depression (AOR = 0.20, 95% CI [0.11–0.38]) and anxiety (AOR = 0.18, 95% CI [0.10–0.34]) compared with those who reported having not enough to make ends meet. Additionally, those who reported having just enough to make ends meet were less likely to have anxiety than those who reported not having enough to make ends meet (AOR = 0.40, 95% CI [0.20–0.78]). Interestingly, family members recruited from rural primary care clinic locations were less likely to have depression (AOR = 0.68, 95% CI [0.48–0.97]) and anxiety (AOR = 0.56, 95% CI [0.37–0.86]) than those recruited from urban primary care clinic locations, while family members recruited from suburban primary care clinic locations were less likely to have anxiety than those recruited from urban primary care clinic locations (AOR = 0.67, 95% CI [0.48–0.92]). Furthermore, our sensitivity analysis revealed no difference in depression and anxiety between family members recruited before and during COVID-19, when adjusting for those who had COVID-19 or lost a family member, friend, or neighbor to COVID-19. 

## 4. Discussion

The researchers found that patients and family members recruited during the COVID-19 pandemic had no difference in depression and anxiety compared with patients and family members recruited before the COVID-19 pandemic. It was encouraging that the rates of depression and anxiety did not change in this population. Additionally, researchers found higher rates of depression and anxiety in patients and family members with specific characteristics who were recruited at both time points. These findings aligned with prior research on what we know about depression and anxiety in older adults and highlight the heightened vulnerability of some groups during crises, such as COVID-19.

Researchers found that patients and family members recruited during the COVID-19 pandemic had no difference in depression and anxiety compared with those recruited before the pandemic. These findings are consistent with a prior cross-sectional study conducted in the Netherlands, which found that while there was both elevated social and emotional loneliness in older adults (aged ≥65 years old) surveyed during the pandemic, mental health remained nearly stable across both timepoints [18]. Additionally, a cross-sectional survey conducted in Germany in April 2020 found no difference in mental health well-being outcomes (depression, anxiety, somatization, and psychological stress) in German older adults (aged ≥65 years old) from prior reported findings from German population-based studies before the COVID-19 pandemic [19]. The consistent rate of mental health among older adults throughout the COVID-19 pandemic is promising; however, different findings were reported by a cross-sectional analysis conducted in the United States (US). Researchers found that the COVID-19 pandemic had negative implications on US older adult mental health, finding a higher incidence of depression during COVID-19 when compared to before the outbreak [20]. These contradictory findings may reflect the study design, as the same older adults who completed a study with similar measures were all contacted to complete follow-up measures during COVID-19. Likewise, analyses conducted by Kaiser Family Foundation compared depression and anxiety among older adults (aged ≥65 years old) who completed the 2018 Centers for Medicare and Medicaid Services Medicare Current Beneficiary Survey (MCBS) to the Census Bureau's Household Pulse Survey 2020 data [21]. The researchers found significantly higher rates of depression and anxiety among US older adults during the COVID-19 pandemic compared with depression and anxiety rates reported in 2018 MCBS. Interestingly, the researchers found a consistent rate of depression and anxiety reported in March 2020 and August 2020, finding that one in four older adults had depression and anxiety. This finding supports no change in the rates of depression and anxiety at two different timepoints of the pandemic. Meanwhile, a longitudinal study among older adults (aged ≥60 years old) found that depressive symptoms increased in Chilean older adults [22], while an observational cohort study found that depressive symptoms more than doubled among Irish older adults during COVID-19 [23]. These findings could be explained by the follow-up analyses performed on the same participants across multiple timepoints. A representative survey panel created by the CDC in June 2020 found that among adults aged 18 years or older, adults aged 65 years or older had significantly lower rates of depression, anxiety, and suicidal ideation compared to the age groups 18–24 years and 25–44 years old [12], indicating that COVID-19 has not affected older adults' mental health as much as younger age groups.

Although we found no significant difference in rates of depression or anxiety, it is important to continue monitoring the mental health of older adults. Moreover, these findings demonstrate the stability of older adults' mental health despite the unprecedented pandemic and increased morbidity and mortality for their age group, which could be supported by prior research findings that older adults report superior emotional regulation and mastery over their emotions [23, 24]. A study of US and Canadian adults found that adults aged ≥60 years old had a lower risk for mental health complications during COVID-19 than their younger counterparts [25]. Along with this, prior research has noted that older adults reported managing their emotions better at their current age compared to when they were younger [24]. This could explain the consistency in rates of depression and anxiety among older adults during the COVID-19 pandemic. Future studies should explore individual characteristics or extraneous factors of older adults that lead to mental health stability, low stress reactivity, coping strategies, and strong emotional regulation. Future research will also want to continue monitoring the mental health status of older adults through the remainder of our current clinical trial to determine whether depression and anxiety may be heightened due to a failure to return to normalcy after COVID-19.

Our study found certain characteristics of patients and family members who had a higher risk of depression and anxiety. Among patients and family members, those who had a comfortable income had significantly lower rates of depression and anxiety compared to those who reported having not enough to make ends meet. Both patients and family members with just enough to make ends meet were less likely to have anxiety compared to those who reported having not enough to make ends meet. Meanwhile, patients with just enough to make ends meet were also less likely to have depression compared to patients who reported having not enough to make ends meet. Additionally, patients with a high school degree or less and patients with some college or college degree were more likely to have anxiety compared with patients with a postgraduate degree. Meanwhile, family members with a high school degree or less were more likely to have depression compared to family members with a postgraduate degree. Furthermore, African American family members were less likely to have depression and anxiety compared to white family members. Increased family member age was also associated with lower depression and anxiety. Our findings are consistent with a prior systematic review that analyzed risk factors for depression and anxiety in older adults during COVID-19 [26]. Additionally, another systematic review consisting of studies of the general population found lower education status to be a risk factor for depression, anxiety, and psychological distress [27]. Moreover, our findings support the notion that certain demographics of older primary care patients and their family members are more at risk for depression and anxiety.

The results of this study support previous research demonstrating stable rates of depression and anxiety in older adults during COVID-19; however, to the best of our knowledge, this study's reported rates of depression and anxiety in the family members of older adults are novel findings. These family members are in a unique position with the designation as the primary support person for older adults. Reported feelings of depression and anxiety among this population may reflect increased responsibility and changes in their personal mental health secondary to COVID-19 and their potential transitioning role as primary caregivers for an older adult [12, 28]. Ideas for future research could include analyzing rates of depression and anxiety in various subgroups of family members (e.g., adult child caregivers, spouses, and friends) and identifying protective characteristics and risk factors in these relationships.

Although not a focus of our analysis, it is possible that media exposure regarding COVID-19 may have some influence on depression and anxiety [29, 30]. Misinformation, lack of information, or excessive information can negatively affect mental health [31, 32].

The limitations of this study include that the results are not causal due to the study design. Different groups of participants were assessed before COVID-19 and during COVID-19. If the same participants were assessed longitudinally before COVID-19 and again during COVID-19, it would allow more direct comparison and the potential for causative conclusions. Additionally, significant differences were observed between the participant characteristics in the cohorts and could be one of the reasons no differences were found, even though we adjusted for these differences in the analysis. Another limitation is the potential impact of a COVID-19 infection on an older adult patient or family member in the COVID-19 cohort. A personal experience with COVID-19, whether an asymptomatic, mild infection or possibly losing a family member or loved one from COVID-19, has the potential to cause significant psychologic distress and impact results [33, 34]. We found that there was no difference in depression or anxiety between the cohorts as before, but this may have been due to the limited reach of COVID-19 to our respondents. For those who were affected by COVID-19, we observed a trend of increasing anxiety and depression. However, that effect was muted by the small number of respondents directly affected by COVID-19. An additional limitation is the end date of data collection. An end date for data collection needed to be set in order to draw conclusions and share our data; however, COVID-19 continued beyond September 2021, and trends in mental health may shift. The world's behavioral and emotional responses to the pandemic are ever-changing, and our results may have varied with a lengthened deadline for data collection. Further, we should consider if those who were more impacted by COVID-19 (e.g., higher depression and anxiety) may have opted to not participate in our study during the COVID-19 pandemic.

## 5. Conclusion

Despite no difference in before- and during-COVID-19 scores on depression and anxiety levels, older primary care patients and their family members still remain vulnerable to mental health issues. Particularly, patients and family members with lower education levels and perceived income are among the most vulnerable. Continual monitoring is necessary.

## Figures and Tables

**Table 1:** Comparison of baseline characteristics.

Variables	Patient			Family member		
	Recruited before COVID-19 *n* = 1118 (%)	Recruited during COVID-19 *n* = 691 (%)	*P*-value	Recruited before COVID-19 *n* = 1118 (%)	Recruited during COVID-19 *n* = 691 (%)	*P*-value
Age in years, mean (SD)	73.8 (6.0)	73.6 (5.1)	0.506	63.6 (13.1)	65.1 (12.6)	0.014
Sex						
Female	589 (52.7)	372 (53.8)	0.633	769 (68.8)	455 (65.8)	0.185
Race						
African American	186 (16.7)	55 (8.0)	<0.001	184 (16.5)	58 (8.4)	<0.001
White	909 (81.5)	627 (90.9)		908 (81.5)	616 (89.5)	
Other race	20 (1.8)	8 (1.2)		22 (2.0)	14 (2.0)	
Ethnicity						
Non-hispanic	1105 (98.8)	681 (98.7)	0.790	1105 (99.0)	685 (99.6)	0.196
Education level						
High school or less	233 (20.8)	96 (13.9)	<0.001	199 (17.8)	75 (10.9)	<0.001
Some college or college degree	607 (54.3)	351 (50.8)		630 (56.4)	401 (58.0)	
Postgraduate	278 (24.9)	244 (35.3)		288 (25.8)	215 (31.1)	
Income			0.037			0.001
Comfortable	878 (79.7)	577 (84.0)		878 (80.3)	589 (87.0)	
Just enough to make ends meet	186 (16.9)	97 (14.1)		181 (16.5)	79 (11.7)	
Not enough to make ends meet	38 (3.4)	13 (1.9)		35 (3.2)	9 (1.3)	
Relationship to the patient						0.019
Spouse or partner				695 (62.2)	477 (69.0)	
Daughter or son				316 (28.3)	162 (23.4)	
Daughter in law or son in law				18 (1.6)	7 (1.0)	
Sibling				40 (3.6)	27 (3.9)	
Other				49 (4.4)	18 (2.6)	
Primary care clinic location			<0.001			
Rural	164 (14.7)	213 (30.8)				
Suburban	554 (49.6)	256 (37.0)				
Urban	400 (35.8)	222 (32.1)				
Mental health status depression			0.041			0.554
None	793 (70.9)	525 (76.0)		875 (78.3)	538 (77.9)	
Mild depression	236 (21.1)	131 (19.0)		190 (17.0)	110 (15.9)	
Moderate depression	62 (5.6)	27 (3.9)		40 (3.6)	32 (4.6)	
Severe depression	27 (2.4)	8 (1.2)		13 (1.2)	11 (1.6)	
Anxiety			0.107			0.084
None	971 (86.8)	619 (89.6)		946 (84.6)	441 (86.0)	
Mild anxiety	106 (9.5)	59 (8.5)		121 (10.8)	74 (10.7)	
Moderate anxiety	22 (2.0)	9 (1.3)		45 (4.0)	15 (2.2)	
Severe anxiety	19 (1.7)	4 (0.6)		6 (0.5)	8 (1.2)	

**Table 2:** Logistic regression results for patient depression and anxiety adjusting for patient characteristics.

Variables	Depression^*∗*^		Anxiety^*∗∗*^	
	Adjusted OR (95% CI)	*P*-value	Adjusted OR (95% CI)	*P*-value
Recruited During COVID	0.84 (0.67, 1.06)	0.134	0.85 (0.62, 1.17)	0.328
Age	0.98 (0.96, 1.00)	0.074	0.98 (0.96, 1.01)	0.159
Sex				
Male	0.78 (0.62, 0.97)	0.025	0.84 (0.62, 1.13)	0.247
Race				
African american	0.82 (0.58, 1.16)	0.273	0.66 (0.41, 1.05)	0.078
White (reference)	1.00		1.00	
Other	0.71 (0.27, 1.86)	0.489	0.24 (0.03, 1.88)	0.175
Education				
High school or less	1.40 (1.00, 1.96)	0.052	2.13 (1.35, 3.35)	0.001
Some college or college degree	1.27 (0.97, 1.65)	0.079	1.48 (1.01, 2.17)	0.047
Postgraduate (reference)	1.00		1.00	
Income				
Comfortable	0.21 (0.11, 0.37)	<0.001	0.23 (0.13, 0.44)	<0.001
Just enough to make ends meet	0.43 (0.23, 0.80)	0.008	0.44 (0.23, 0.85)	0.015
Not enough to make ends meet (reference)	1.00		1.00	
Recruitment Setting				
Rural	0.72 (0.52, 1.00)	0.049	0.72 (0.46, 1.13)	0.152
Suburban	0.98 (0.75, 1.26)	0.849	0.86 (0.61, 1.22)	0.398
Urban (reference)	1.00		1.00	

Note. ^*∗*^(PHQ-9 score of ≥5); ^*∗∗*^(GAD-7 score ≥5).

**Table 3:** Logistic regression results for family member depression and anxiety adjusting for family member characteristics.

Variables	Depression^*∗*^		Anxiety^*∗∗*^	
	Adjusted OR (95% CI)	*P*-value	Adjusted OR (95% CI)	*P*-value
Recruited During COVID	1.18 (0.92, 1.52)	0.187	1.04 (0.78, 1.40)	0.776
Age	0.99 (0.98, 1.00)	0.009	0.97 (0.96, 0.98)	<0.001
Sex				
Male	0.84 (0.65, 1.10)	0.203	0.76 (0.55, 1.05)	0.097
Race				
African American	0.52 (0.35, 0.78)	0.001	0.57 (0.37, 0.89)	0.013
White (reference)	1.00		1.00	
Other	1.68 (0.81, 3.48)	0.165	0.78 (0.29, 2.12)	0.629
Education				
High school or less	1.47 (1.01, 2.15)	0.047	1.54 (0.98, 2.42)	0.061
Some college or college degree	1.25 (0.94, 1.67)	0.126	1.36 (0.96, 1.93)	0.085
Postgraduate (reference)	1.00		1.00	
Income				
Comfortable	0.20 (0.11, 0.38)	<0.001	0.18 (0.10, 0.34)	<0.001
Just enough to make ends meet	0.54 (0.28, 1.04)	0.064	0.40 (0.20, 0.78)	0.007
Not enough to make ends meet (reference)	1.00		1.00	
Recruitment Setting				
Rural	0.68 (0.48, 0.97)	0.031	0.56 (0.37, 0.86)	0.007
Suburban	0.86 (0.65, 1.13)	0.274	0.67 (0.48, 0.92)	0.014
Urban (reference)	1.00		1.00	

*Note.* ^*∗*^(PHQ-9 score of ≥5); ^*∗∗*^(GAD-7 score ≥5).

## Data Availability

The dataset used to support the findings of this study are available from the corresponding author upon request.
